# Immunomodulatory effects of a mycelium extract of Cordyceps (Paecilomyces hepiali; CBG-CS-2): a randomized and double-blind clinical trial

**DOI:** 10.1186/s12906-019-2483-y

**Published:** 2019-03-29

**Authors:** Su-Jin Jung, Eun-Soo Jung, Eun-Kyung Choi, Hong-Sig Sin, Ki-Chan Ha, Soo-Wan Chae

**Affiliations:** 1 Clinical Trial Center for Functional Foods, Chonbuk National University Hospital, Jeonju, Republic of Korea; 2 Chebegen Inc., Jeonju, Republic of Korea; 3 Healthcare Claims & Management Incorporation, Jeonju, Republic of Korea; 4 Biomedical Research Institute, Chonbuk National University Hospital, Jeonju, Republic of Korea; 5 Department of Pharmacology, Chonbuk National University, Medical School, 567 Baekje-daero, Deokjin-gu, Jeonju, 54896 Republic of Korea

**Keywords:** Cordyceps, Immune, Natural killer (NK) cells, Mycelium culture, *Paecilomyces hepiali*

## Abstract

**Background:**

Cordyceps is a traditional Chinese herb that produces various biopharmaceutical effects, including immune-enhancing effects. In this study, we prepared a Cordyceps mycelium culture extract (*Paecilomyces hepiali,* CBG-CS-2) to confirm its efficacy in enhancing the immune system and to evaluate its safety in healthy adults.

**Methods:**

Healthy adults were divided into the intervention group (*n* = 39), who were given 1.68 g/day of CBG-CS-2 in capsules, and the control group (*n* = 40) for 8 weeks. The activities of natural killer (NK) cells and serum levels of monocyte-derived mediators were assessed initially for a baseline measurement and after 8 wks.

**Results:**

The CBG-CS-2 group showed a significant 38.8 ± 17.6% enhancement from the baseline of NK cell cytotoxic activity relative to the placebo group after the administration of the capsules for 8 wks. (*P < 0.019*).

**Conclusion:**

The results suggest that the immune system functions well with CBG-CS-2 supplementation, perhaps with less accompanying inflammation. Thus, CBG-CS-2 is safe and effective for enhancing cell-mediated immunity in healthy adults.

**Trial registration:**

This study was registered at Clinical Trials.gov (NCT 02814617).

**Electronic supplementary material:**

The online version of this article (10.1186/s12906-019-2483-y) contains supplementary material, which is available to authorized users.

## Background

Cordyceps is a natural substance that has traditionally been used as a Chinese medicine in East Asian countries. In particular, *Cordyceps sinensis* (CS) is largely known as a traditional Chinese medicine [1]. However, its use as a popular medicine is limited due to difficulties in the mass gathering of natural CS. It is a type of wild mushroom grown at 3000~4000 m above sea level around the Himalaya Mountains in Tibet and the high regions in China, and until recently, it was impossible to artificially cultivate due to the difficult growth conditions [2]. In recent years, however, it has become possible to artificially cultivate CS through the development of artificial production technologies that overcome both the decrease in Cordyceps supply and the heterogeneity in its composition [3–5]. Recently, there have been some empirical studies on the effectiveness of mass-produced Cordyceps, and several trials have been conducted in various fields, such as medicine, cosmetics, and functional foods [4, 6–9]. The mycelial cultured CS mycelia (*P. hepiali*) products include strong bioactive substances, such as nucleosides and polysaccharides (PS), which are part of the bioactive substances of natural CS. Thus, it has been recognized that the bioactivities of mycelial cultured CS are very similar to those of natural Cordyceps [10–13]. In general, the *Paecilomyces hepiali* (*P. hepiali*) that is commonly included in natural CS from Tibet is known as an endoparasitic fungus. The genome sequence of *P. hepiali* is the medical compound produced using fungi, and there are some trials where it is being applied and developed in different fields. The main components of CS, such as polysaccharides, cordycepin, adenosine, cordycepic acid, nucleosides, and ergosterol, are known to be important bioactive substances with medical relevance [14–16]. There have been several reports on the health functionality of these bioactive substances, which include important functions such as immunomodulatory, anticancer, antimetastatic, anti-oxidant, anti-inflammatory, antimicrobial, lipid metabolism improvement, hypoglycemic, anti-aging, and liver and renal recovery functions [11, 17–29].

In our preclinical study, animal testing in an immunosuppressed mouse model (mitomycin C-treated mice; MMC) verified the immunomodulatory effect of a mycelium extract from *P. hepiali* isolated from CS (CBG-CS-2) [30, 31]. We also verified that the extract helped reduce a gut immunity suppressive state by controlling the expression of the immune response genes and increased both the NK-cell activity and production of nitric oxide (NO). In addition, the effects of controlling the anti-oxidant, antifatigue, and anti-inflammatory functions, including immunomodulation, were identified by removing free radicals based on the suppression of the excessive expression of inducible nitric oxide synthase (iNOS) and the excessive production of the TNF-α inflammatory mediators [30–32]. However, in our preclinical studies, the effectiveness and safety of CBG-CS-2 for an immunoregulatory effect were not evaluated, although the mechanism and effectiveness of the immunomodulating effect of CBG-CS-2 separated and cultivated from *P. hepiali* from CS were verified.

The objectives of this study were to confirm the immunoregulatory efficacy and safety of CBG-CS-2 separated and cultivated from *P. hepiali* from CS in healthy Korean adults.

## Methods

### Participants

The study subjects were recruited and selected at the Clinical Trials Center for Functional Foods (CTCF2) at Chonbuk National University Hospital from September to November 2015. A total of 80 healthy male and female subjects agreed to participate in this study. A total of 80 participants were randomly assigned into one of the study groups (40 subjects each) using a computer-generated random number table by the Randomization program of the version 9.2 SAS® system (SAS Institute, Cary, NC, USA).

The criteria for the selection and exclusion of participants in this study are described below.

The selection guidelines were as follows:

1) Male and female adult participants aged 20~75 at the time of the screening test.

2) Participants who showed a slight decrease in immunity and had a peripheral blood white blood cell (WBC) level of 3*10^3^ ~ 7*10^3^ cells/μl measured during the screening test.

3) Participants who fully understood the test and decided to participate of their own free will and agreed to the written consent document.

The exclusion criteria were as follows:

1) Male and female adult participants who had a BMI less than 18.5 kg/m^2^ at the time of the screening test.

2) Participants who had a family history of medicinally or clinically significant hypersensitivity reactions.

3) Participants with thyroid or hypophyseal disorder.

4) Participants with acute severe cardiovascular diseases such as cardiac insufficiency, myocardial infarction, or stroke.

5) Participants with immunological disease, liver or renal failure.

6) Participants who had specific diseases such as a malignant tumor, lung disease, leukemia, collagenosis, multiple sclerosis, allergic skin disease, and other autoimmune disorders or a history thereof.

7) Participants diagnosed with diabetes.

8) Participants who had a “gastroesophageal reflux disease”, such as Crohn’s disease.

9) Participants who, within 2 weeks from the first intake day, had ingested medicine, Chinese medicine, or a health functional food that can affect the immunomodulatory effect of the test product. In the case of health functional foods, it was possible to participate in this program after a one-week wash out period.

10) Participants who had a history of treatment with anti-psychotics within 2 months of the screening test.

11) Participants who had a history of treatment for alcoholism or drug abuse.

12) Those who participated in other clinical studies within 2 months prior to the date of the first clinical test product intake.

13) Participants who had an alanine transaminase (ALT) or aspartate transaminase (AST) plasma level more than three times the guideline of the organization.

14) Female participants who were pregnant or breastfeeding or planned to become pregnant during the test period.

15) Participants who had disqualifying results in the diagnostic test and were inappropriate for other reasons.

All subjects gave written informed consent before entering the study. The study was conducted according to the Declaration of Helsinki. The study protocol was approved by the Functional Food Institutional Review Board (FFIRB) of Chonbuk National University Hospital (FFIRB number 2015–02-010) on August 25, 2015. This study was registered on June 28, 2016 after the termination of the study sub heading: The efficacy and safety of CS mycelium culture extract (*P. hepiali*, CBG-CS-2) on the promotion of immunity.

### Study design

This study was an 8 week, randomized, double blind, and placebo-controlled clinical trial that was performed according to a computer-generated randomization schedule designed to assign subjects to the CBG-CS-2 or placebo group. Neither the investigators nor the subjects knew the randomization code or the results of the NK cell activity levels until after statistical analysis was complete. Subjects attended a screening visit (within 4 weeks), at which inclusion and exclusion criteria were assessed. The enrolled subjects were scheduled for their first visit, and subjects were randomly assigned to one of two groups, either the CBG-CS-2 (*n* = 40) or placebo group (*n* = 40). Subjects received either the CBG-CS-2 or placebo capsules every week, and all of the subjects were instructed to take either two CBG-CS-2 capsules (twice per day) or two placebo capsules (twice per day) per day (1.68 g/day) after breakfast and dinner for 8 weeks.

During the intervention period of 8 weeks, subjects were asked to continue their usual diets and activity and to not ingest any other functional foods or dietary supplements. Anthropometric and biochemical parameters, vital signs, and nutrient intake were measured before and after the intervention period. Every week, the subjects were asked to report any adverse events or changes in training, lifestyle, or eating patterns and to assess their capsule-dosing compliance.

A CONSORT checklist for the reporting of this study can be found in Additional file 1.

### Preparation of test materials

Cordyceps mycelium extract (*Paecilomyces hepiali,* CBG-CS-2) was provided by Chebeigen, Inc. (Jeonju, Republic of Korea), and CS mycelium culture extracts were prepared as described previously [33]. The main components of the mycelium culture extract were as follows: 32% cordyceps polysaccharide, 7.3% cordycepic acid, 0.13% adenosine, and 0.001% cordycepin [33]. Main component profiles in the CBG-CS-2 were analyzed by high-performance liquid chromatography (HPLC) according to the Korean Food and Drug Administration (KFDA) guidelines, and these cordyceps polysaccharide profiles are shown in (Table 1). The extract was administered as a dark brown powder composed of 85.36% CS mycelium culture extract powder peel, 12.64% microcrystalline cellulose, and 2.0% hydroxylpropyl methylcellulose. The placebo was composed of 84.0% microcrystalline cellulose, 14.2% hydroxylpropyl methylcellulose, and 0.6% orange yellow.

**Table 1:** Composition of the test product

Component	Test capsule Cordyceps mycelium extract powder supplement (%)	Placebo supplement (%)
Cordyceps mycelium extract powder	85.36	–
Microcrystalline cellulose	12.64	84.0
Hydroxylpropyl ethyl cellulose	2.0	14.2
Cacao coloring	–	0.5
Caramel coloring	–	0.3
orange yellow	–	0.6
Red cabbage color	–	0.4
Total	100	100

Determined by high-performance liquid chromatography (HLPC)

### Biochemical analyses

The efficacy evaluation and safety evaluation parameters before the baseline (Week 0) of the human study and after eight weeks of participation were analyzed prior to participation in this human study for all of the subjects. The fasting blood samples of all subjects were collected and centrifuged (Hanil Science Industrial Co., Ltd. Seoul, Korea) at 3000 rpm for 20 min while keeping an empty stomach for more than 12 h from the day before the blood collection, and they were kept frozen at − 80 °C until the analysis. The blood samples were analyzed using a Hitachi 7600–110 analyzer (Hitachi High-Technologies Corporation, Tokyo, Japan). The primary outcomes of this clinical trial measured the changes in natural killer cell activity [28, 34], and the secondary outcomes measured cytokines (IFN-γ and TNF-α, IL-1β, IL-2, IL-4, IL-10, and IL-12). The assessment of the safety parameters was performed through measuring an electrocardiogram (ECG) as well as laboratory tests **(**WBCs, RBCs, hemoglobin, hematocrit, platelets, total bilirubin, γ-glutamyl transferase (GGT), ALT, AST, alkaline phosphatase (ALP), TC, TG, LDL-C, HDL-C, fasting blood glucose, total protein, albumin, blood urea nitrogen (BUN), creatinine, creatine kinase, lactate dehydrogenase, Na, K, Cl, Ca, and P). Vital signs (systolic blood pressure, diastolic blood pressure, and pulse) of the study subjects were measured at every visit.

### Statistical analysis

All statistical processing was analyzed using SAS version 9.2 (SAS Institute, Cary, NC, USA). All data were expressed as the mean ± standard deviation (SD) for continuous variables and as a frequency (%) for categorical variables. In this study, the intention-to-treat (ITT) population visited at least once and included performing an analysis of the efficacy and safety of those who had undergone measurements on the main evaluation variables after taking the CBG-CS-2 and placebo products. The data on the efficacy evaluation were based on the per-protocol (PP) group as the main analysis target, but a further analysis of the ITT group was implemented for its efficacy. To detect a 7.4% (SD 11.4%) difference in NK-cell activity change between groups with a power of 80% with a two-tailed alpha level of 0.05, a sample size of 40 per group (*N* = 80) was needed [35]. In the case of the categorical variables, the significance test was applied with the Chi-square test (Fisher’s exact test). Additionally, the baseline and endpoint comparison of the investigation results of the evaluation variables in each group were tested by applying the paired t-test. The mean comparison between the two groups was tested with the independent sample t-test for independent samples, and the outcome variables for repeated measurement of the intake groups were applied with a linear mixed model within the intake groups and between the intake groups. The significance was statistically significant at the level of *p* < 0.05.

## Results

### Demographic and clinical characteristics of participants

**Fig. 1:**
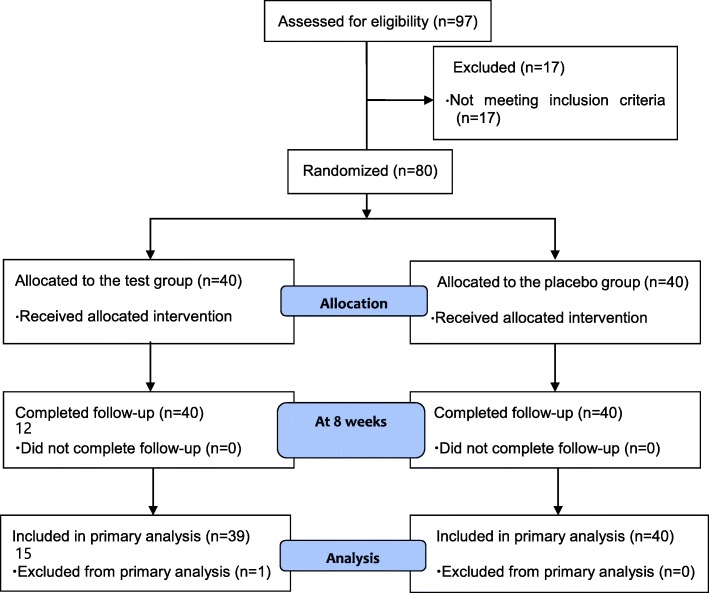
Study flow diagram based on the CONSORT 2010 flow diagram

Ninety-seven participant were assessed for eligibility, and a total of 80 participants (mean age = 47.6 ± 5.9 years, 52 males and 28 females, mean body mass index = 24.3 ± 3.0 kg/m^2^, NK-cell activity mean = 35.8 ± 17.7%) met the inclusion criteria. Seventeen participants were excluded from the current analysis because they did not meet all of the inclusion criteria. Thus, a total of 79 participants completed the study (Fig. 1). One subject from the CBG-CS-2 group failed to complete the study. One subject was excluded because of the lack of compliance. As a result, 79 subjects (CBG-CS-2 group = 39 and placebo group = 40) remained. There were no significant differences between groups in the baseline characteristics such as age, sex, height, weight, body mass index, NK cell activity, IL-1ß, IL-2, 4, 10, 12, INF-γ, TNF-α, alcohol intake, and smoking status (Table 2). The compliance rates, which were based on pill count, were 94.5 ± 7.0% and 94.6 ± 6.1% in the placebo and CBG-CS-2 groups, respectively.

**Table 2:** Study participant demographic characteristics

	Cordyceps mycelium extract group (*n* = 40)	Placebo group (*n* = 40)	Total (*n* = 80)	*P*-value^a^
Age (years)	48.0 ± 6.3	47.3 ± 5.7	47.6 ± 5.9	0.563
Sex (M/F)	25/15	27/13	52/28	0.639
Height (cm)	167.6 ± 8.4	165.9 ± 7.3	166.7 ± 7.9	0.344
Weight (kg)	66.7 ± 9.9	68.7 ± 12.0	67.7 ± 11.0	0.405
BMI (kg/m^2^)	23.7 ± 2.7	24.8 ± 3.1	24.3 ± 3.0	0.084
NK-cell activity (25:1) (%)	33.7 ± 17.4	37.9 ± 18.2	35.8 ± 17.7	0.296
IL-1ß (pg/mL)	3.1 ± 1.3	4.0 ± 7.7	3.5 ± 5.5	0.505
IL-2 (pg/mL)	3.0 ± 0.8	2.9 ± 0.6	3.0 ± 0.7	0.385
IL-4 (pg/mL)	10.4 ± 2.7	11.0 ± 6.8	10.7 ± 5.2	0.652
IL-10 (pg/mL)	5.1 ± 4.5	3.8 ± 3.0	4.4 ± 3.8	0.139
IL-12 (pg/mL)	5.8 ± 6.1	5.1 ± 3.3	5.4 ± 4.9	0.537
INF-γ (pg/mL)	10.5 ± 5.5	10.0 ± 5.1	10.2 ± 5.2	0.625
TNF-α (pg/mL)	6.5 ± 1.4	6.2 ± 1.5	6.3 ± 1.5	0.339
Drinker (person)	25 (62.5%)	19(47.5%)	44(55.0%)	0.178
Drinking quantity (units)	3.3 ± 2.9	4.7 ± 4.5	3.9 ± 3.7	0.235
Smoker (person)	4(10.0%)	4(10.0%)	8(10.0%)	> 0.999
Smoking amount (cigarettes)	13.0 ± 4.8	17.5 ± 5.0	15.3 ± 5.1	0.240

Values are presented as the mean ± SD or number (%)

^a^ Analyzed by an Independent t-test, Chi-square test or Fisher’s exact test

### Safety analysis

Safety outcomes were assessed by assessing adverse events during the overall clinical study period. The laboratory tests (blood and urinalysis), ECG, vital signs, and anthropometric parameters (data not shown) were evaluated. At every visit, information about symptoms or adverse events was recorded, but no serious adverse events were reported during the study period. The parameters of the safety assessments were in the normal range, and no subjects withdrew because of adverse events.

### Efficacy evaluation

Changes in the NK-cell activity and cytokines (IFN-γ, TNF-α, IL-1β, IL-2, IL-4, and IL-10, and IL-12) during the overall intervention period are shown in Table 3.

**Table 3:** NK-cell activity and cytokine cluster analysis obtained prior to and after treatments from the two groups

	Cordyceps mycelium extract group (*n* = 39)			Placebo group (*n* = 40)			*p*-value^b^
	Baseline	Week 8	*p*-value^a^	Baseline	Week 8	*p*-value^a^	
NK-cell activity (%)	33.7 ± 17.4	38.8 ± 17.6	0.025^*^	37.9 ± 18.2	35.5 ± 19.9	0.282	0.019^*^
IL-1ß (pg/mL)	3.1 ± 1.3	3.1 ± 2.0	0.741	4.0 ± 7.7	2.7 ± 0.7	0.328	0.360
IL-2 (pg/mL)	3.0 ± 0.8	3.0 ± 0.7	0.446	2.9 ± 0.6	3.0 ± 0.5	0.166	0.126
IL-4 (pg/mL)	10.3 ± 2.7	10.2 ± 2.2	0.662	11.0 ± 6.8	10.9 ± 7.8	0.979	0.764
IL-10 (pg/mL)	5.1 ± 4.5	6.1 ± 6.3	0.205	3.8 ± 3.0	4.2 ± 3.0	0.277	0.458
IL-12 (pg/mL)	5.8 ± 6.2	6.0 ± 6.3	0.389	5.1 ± 3.3	5.2 ± 3.3	0.766	0.523
INF-γ (pg/mL)	10.4 ± 5.4	11.4 ± 7.1	0.100	10.0 ± 5.1	10.7 ± 6.7	0.275	0.741
TNF-α (pg/mL)	6.5 ± 1.5	6.8 ± 1.5	0.214	6.2 ± 1.5	6.6 ± 2.0	0.114	0.918

Values are presented as the mean ± SD

^*^p < 0.05

^a^Analyzed by a paired t*-*test within groups

^b^Analyzed by a linear mixed effect model for repeated measurement data between groups

The rate of change in NK-cell activity increased up to 38.8 ± 17.6% in the CBG-CS-2 group but decreased by 35.5 ± 19.9% in the placebo group. A significant difference between these two groups was observed *(P = 0.019)*. Additionally, the CBG-CS-2 and placebo groups showed increases in the level of IFN-γ of 11.4 ± 7.1% and 10.7 ± 6.7%, respectively. However, there was no significant difference between these two groups. In addition, with respect to the levels of TNF-α and IL-12, the CBG-CS-2 group showed a tendency toward increasing levels, but there was no significant difference between the two groups. Furthermore, there were no significant differences in the levels of other cytokines such as IL-1β, IL-2, IL-4, and IL-10.

## Discussion

In this study, we performed an 8-week clinical trial to evaluate the immunoregulatory function and safety of a mycelium extract of Cordyceps (CBG-CS-2; 1.43 g/day) separated and cultivated from *P. hepiali* derived from natural CS picked in Tibet. In healthy Korean test subjects, the NK-cell activity increased significantly after CBG-CS-2 was taken for 8 weeks. In the CBG-CS-2 group, the changes in NK-cell activity represented a higher immune regulatory effect than that observed in the placebo group. Additionally, we verified that CBG-CS-2 had an immune pharmacology efficacy in our in vitro and in vivo tests. In particular, the oral administration of CBG-CS-2 for 28 days in an immune-suppressed mouse model (mitomycin C-treated mice: MMC) helped reduce a gut immunity suppressive state in Peyer’s patches [33] and increased splenocyte proliferation in C57BL6 mice. Furthermore, we verified that it increased the generation of IL-12, which is an important cytokine in the T-helper cell 1 (Th1 cell) reaction related to cell-mediated immunity. In addition, it can activate the release of interferon (IFN)-γ through activating immunocytes such as dendritic cells and macrophages and has a clear immune regulatory effect caused by increasing tumor necrosis factor-alpha (TNF-α) and enhancing NK-cell activity [30, 31]. It is believed that this mechanism promotes the proliferation of splenocytes caused by the administration of CBG-CS-2 and that this increases the expression of IL-12, IFN-γ, and TNF-α by stimulating a T-helper cell-type immune reaction in mouse splenocytes. In addition, the extract improved cell-mediated immunity by increasing the NK-cell activity [31]. However, there were significant changes in the serum cytokines, including IL-12 and IFN-γ, in the clinical trial compared with our previous animal test, in which only the NK-cell activity was increased, that is, the NK-cell activity increased without any changes in the levels of cytokines. This might be due to the measurement focusing on changes in the activity of immune reactions after applying the K562 cells to trigger an immune reaction in peripheral blood mononuclear cells (PBMCs) by separating the immunocytes from blood. Kang et al. [28] showed that the intake of *C. militaris* for 4 weeks by healthy adults increased both the NK-cell activity and levels of the cytokines IL-12 and IFN-γ; these findings differ from the results of this study. It is known that NK-cells in the human body play a role in innate immunity by detecting and killing virus-infected cells, tumor cells, and abnormal cells and that activated NK-cells promote the release of cytokines such as IFN-γ and TNF-α [36, 37]. Kuo et al. [38] observed that in healthy adults, the processing of LPS in immunocytes (BALF cells) sampled from bronchial tubes after taking CS decreased Th2 cytokines and increased the release of Th1 cytokines (IL-12 and IFN-γ). This dynamic controls immune functions based on the balance between Th1 and Th2 cytokines. Additionally, they reported that in healthy people and leukemia patients, the application of Cordyceps increased only the activity of NK-cells [39]. These results are similar to those obtained in the current study. This finding of a change only in NK-cell activity might be because our study investigated changes in serum cytokine indices in healthy participants without any external stimulation or triggering factors. In addition, because it plays a role in maintaining homeostasis in healthy human subjects, it is believed that the extract does not affect cytokine levels [27]. Shin et al. [14] and Kang et al. [28] showed that the major components of *C. militaris*, cordycepin and adenosine, contributed to controlling both the phenotype transition of macrophages as well as the inflammatory and immunomodulatory effects. It is expected that the major components of CBG-CS-2, Cordyceps polysaccharides and adenosine, positively contributed to the immunoregulatory effects even though the extract showed a low cordycepin content compared to that of *C. militaris*. Additionally, it has been shown that simple and protein-bound polysaccharides (PS) separated from Cordyceps had excellent immunomodulatory effects [40, 41]. In particular, it was reported that PS played a role in increasing innate immune and cell-mediated immune responses as a polymer [29, 42]. Additionally, these compounds showed immunomodulatory and tumor growth inhibition effects by increasing the phagocytic activity of macrophages, the proliferation of splenocytes, and the levels of NK-cell activity, IFN-γ, and TNF-α**.** Therefore, we believe that the major components of CBG-CS-2 in this study, Cordyceps PS and adenosine, play an important role in presenting immune reactions as a trigger and induce an immunomodulatory effect by enhancing both the NK-cell activity and phagocyte reactions via the activation of macrophages.

There are some limitations to this study. We likely did not find differences between the CBG-CS-2 and placebo groups in the immune index of the Th1 cytokine cluster because the cytokine index was measured without any triggering factors, which can cause specific immune reactions. Additionally, we used blood samples from healthy adult participants who had no specific diseases.

This study was registered at Clinical Trials.gov on June 2016 (NCT 02814617).

## Conclusion

It will be necessary to investigate changes in immune-related cytokines in participants with depressed immune responses and to precisely design a challenge test that can externally stimulate immune depression. Furthermore, a long-term study will also be required. In summary, it is expected that the continuous dose of CBG-CS-2 obtained from CS increases Th1 immune responses and activates NK-cells, which results in immunomodulatory effects by improving cell-mediated immunity.

## Additional file

Additional file 1: Supplements test capsules. (DOCX 49 kb)

## Abbreviations

CBG-CS-2: Mycelium extract of Cordyceps
CS: Cordyceps sinensis
iNOS: Inducible nitric oxide synthase
MMC: Mitomycin C-treated mice
NK-cell: Natural killer
*P. hepiali*: Paecilomyces *hepiali*
PS: Polysaccharides
