# Supplementary figures and images for: Immunomodulatory effects of a mycelium extract of Cordyceps (Paecilomyces hepiali; CBG-CS-2): a randomized and double-blind clinical trial
Source: BMC Complement Altern Med. 2019 Mar 29;19:77. doi: 10.1186/s12906-019-2483-y (PMC6441223; doi:10.1186/s12906-019-2483-y)

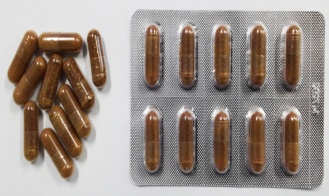


<Supplements of test capsules>

Supplement: Supplementary file 1 — Supplements test capsules. (DOCX 49 kb) [file 12906_2019_2483_MOESM1_ESM.docx]
